# Structure-Based Virtual Screening of Novel Natural Alkaloid Derivatives as Potential Binders of *h-telo* and *c-myc* DNA G-Quadruplex Conformations

**DOI:** 10.3390/molecules20010206

**Published:** 2014-12-24

**Authors:** Roberta Rocca, Federica Moraca, Giosuè Costa, Stefano Alcaro, Simona Distinto, Elias Maccioni, Francesco Ortuso, Anna Artese, Lucia Parrotta

**Affiliations:** 1 Dipartimento di Scienze della Salute, Università degli Studi “Magna Græcia”, Campus “S. Venuta”, Viale Europa, Germaneto 88100, Catanzaro, Italy; E-Mails: rocca@unicz.it (R.R.); fmoraca@unicz.it (F.M.); gcosta@unicz.it (G.C.); ortuso@unicz.it (F.O.); artese@unicz.it (A.A.); lparrotta@unicz.it (L.P.); 2 Dipartimento di Scienze della Vita e dell’Ambiente, Università degli Studi di Cagliari, Via Ospedale 72, Cagliari 09124, Italy; E-Mails: s.distinto@unica.it (S.D.); maccione@unica.it (E.M.)

**Keywords:** DNA, G-quadruplex, *h-telo*, *c-myc*, alkaloids, berberine, virtual screening, docking, molecular dynamics

## Abstract

Several ligands can bind to the non-canonical G-quadruplex DNA structures thereby stabilizing them. These molecules can act as effective anticancer agents by stabilizing the telomeric regions of DNA or by regulating oncogene expression. In order to better interact with the quartets of G-quadruplex structures, G-binders are generally characterized by a large aromatic core involved in π-π stacking. Some natural flexible cyclic molecules from Traditional Chinese Medicine have shown high binding affinity with G-quadruplex, such as berbamine and many other alkaloids. Using the structural information available on G-quadruplex structures, we performed a high throughput *in silico* screening of commercially available alkaloid derivative databases by means of a structure-based approach based on docking and molecular dynamics simulations against the human telomeric sequence *d*[AG_3_(T_2_AG_3_)_3_] and the *c-myc* promoter structure. We identified 69 best hits reporting an improved theoretical binding affinity with respect to the active set. Among them, a berberine derivative, already known to remarkably inhibit telomerase activity, was related to a better theoretical affinity* versus* *c-myc*.

## 1. Introduction

Non-canonical high-order DNA structures, known as G-quadruplexes (G4s), are formed by guanine-rich DNA sequences and are prevalent in the human genome. Bioinformatics analyses show that G4s sequence motifs are present in many important regions of the eukaryotic genome, such as telomere ends and the regulatory regions of several oncogenes, including *c-myc*, *c-kit*, V-Ki-ras2 Kirsten rat sarcoma viral oncogene homolog (KRas), platelet-derived growth factor subunit A (PDGF-A), vascular endothelial growth factor (VEGF), intron regions and the insulin-related polymorphism regions [1,2,3,4,5,6,7,8,9,10,11]. Recently G4s, that demonstrated an important role* in vivo* [12,13,14], were considered as potential targets for cancer therapy [15,16,17].

The presence of monovalent cations, such as Na^+^ and K^+^, and a network of Hoogsteen hydrogen bonds are responsible for the stabilization of the stacked G-tetrad planes that form the G4 structures. Polymorphism is an essential feature of the G4 DNA that assumes various conformations, depending on the counterion type, incubation period and sequence [18]. In particular, the G4s strands intertwine to form parallel, anti-parallel or mixed hybrid conformations which result in a variety of different grooves and loops [19,20,21,22]. The presence of dimeric G4 structures, besides the formation of monomeric intermolecular and intramolecular conformations, is also reported. Specifically, the telomeric G4 DNA can either occur by dimerization of hairpin loops or by directly folding into intramolecular structures [23,24]. Regarding the importance of G4 structures, specific ligands that can induce their formation and stabilization are considered as potential drugs due to their inhibitory action on telomerase activity and oncogene expression [25,26]. The telomerase enzyme, normally absent in somatic cells but present in 85%–90% of cancer cells, is able to add the telomeric repeat unit (TTAGGG in case of humans) to the ends of the chromosome contributing to their immortality [27,28,29]. In order to achieve such an aim, the telomeric region of the chromosome must remain single stranded [30]. Thus, by either blocking telomerase directly or by making the telomerase substrate inaccessible, the uncontrolled cellular proliferation can be stopped. The most interesting therapeutics are small molecule inhibitors, antisense oligonucleotides, immunotherapies, gene therapies, telomere and telomerase associated protein inhibitors, T-oligo and G4 stabilizers. In particular, although there are various selective synthetic and natural G4 molecules, with different chemical scaffolds, widely studied also by our research group [31,32,33,34,35,36,37,38], three of the most commonly investigated G4 stabilizing agents are the trisubstituted acridine BRACO-19, the polycyclic compound RHPS4 and the natural ligand telomestatin [39,40].

In the human genome there are ~376,000 potential G4 forming motifs, as shown by bioinformatics studies [9,11]. These motifs, abundant in the promoter region of genes especially near the transcription start site (*c-myc*, *c-kit*, *c-myb*, KRAS, PDGF-A, VEGF, BCL-2, MyoD, HIF-1α and TK1) suggested a key role of G4 structures in transcription [41]. Among these, the quadruplex in the promoter region of *c-myc* is widely investigated. A variety of malignant tumors are associated with the aberrant over-expression of *c-myc* [42]. This region contains a cytosine-rich (C-rich) coding strand and a guanine-rich (G-rich) non-coding strand that are capable of engaging in a slow equilibrium between B-form duplex DNA, single-stranded DNA, and tetra-stranded DNA. Specifically, the cytosine tracts in the complementary C-rich strand can form an i-motif or i-tetraplex, whereas the guanine tracts of the G-rich strand can form a G4 structure [43]. The nuclear hypersensitivity element III_1_ (NHE III_1_), upstream of the P1 promoter of *c-myc*, controls 80%–90% of the *c-myc* transcription level [44,45]. The NHE III_1_, a G-rich strand of the DNA containing 27 base-pair sequence, is able to form intramolecular G4 structure and functions as a transcriptional repressor element [43,46,47]. The first intramolecular G4 structure within the *c-myc* NHE III_1_ region was proposed by Simonsson* et al.* [45] that described an antiparallel-stranded structure involving three G-tetrads formed from four G-tracts linked by two lateral loops and a central diagonal loop. However, in the *c-myc* NHE III_1_ region further studies, carried out by chemical footprinting, circular dichroic (CD) and nuclear magnetic resonance, revealed that there is a single G4 isomer of parallel topology containing three lateral loops (a 1:2:1 loop isomer) [48]. The G4 present in the NHE III_1_ region of the *c-myc* promoter was shown to function as a silencer element [46]. Consequently, the approach to design specific inhibitors able to stabilize this structure could potentially be an effective approach of targeting human malignancies that overexpress *c-myc*. In fact, Grand and collaborators showed that the cationic porphyrinTMPyP4 decreases *c-myc* expression at both the mRNA and protein levels and reduces the level of several *c-myc*-regulated genes [49]. Moreover, different natural products derived from Traditional Chinese Medicine have been found associated to high binding affinities* versus* diverse G4 sequences, such as berbamine, which is a natural alkaloid able to better stabilize (GGA)_8_ G4 compared with other six G4s [50].

Several studies related to the rational drug design of natural molecules have been performed by means of structure-based techniques. In particular, this approach, complementing conventional high-throughput techniques, represents an efficient strategy in drug discovery [51,52,53,54,55,56,57]. The number of compounds synthesized and tested* in vitro* can be dramatically reduced through the identification of inactive non-binders *in silico*. Meanwhile, an important source of bioactive substructures and chemical scaffolds can be found in natural products [58].

Recently, Gosh* et al.* applied absorption and fluorescence studies, demonstrating that sanguinarine, a naturally occurring plant alkaloid isolated from *Sanguinaria canadensis* [59], had high binding affinity towards two quadruplex forming sequences, human telomeric DNA (H24) and NHE III_1_ upstream of the P1 promoter of *c-myc* (Pu27). Specifically, by means of differential scanning calorimetry studies, sanguinarine binding was found to increase the melting point and also the total enthalpy of transition of both quadruplexes, while TRAP results showed that it effectively blocked telomerase activity in a concentration dependent manner [60].

Sanguinarine and other 10 alkaloids were investigated by Ji* et al.* for their ability to interact with G4 formed by telomeric DNA and *C-myc*22 sequences through molecular modeling and biophysics experiments. In particular, sanguinarine, palmatine and berberine were observed to induce the formation of G4 as well as to stabilize it due to the presence of unsaturated ring, positively charged nitrogen centers, and conjugated aromatic rings [61].

Berberine, an alkaloid isolated from Chinese herbs, was initially used as anti-microbial agent; nowadays it represents a hot research topic, since it demonstrates various pharmacological activities that have applications in several therapeutic areas, such as cancer, diabetes, depression, cardiovascular, and hypertension [62,63,64]. In 2012, Bessi and co-workers, by means of fluorescence, CD, molecular modeling and NMR studies, demonstrated the interactions of the natural alkaloids berberine and sanguinarine with basket- and hybrid-type *h-telo* G4 DNA with a high stoichiometry as a consequence of specific binding modes [65].

Recently, 13-substituted berberine derivatives were reported by Neidle’s group for their abilities to selectively bind to G4 over double-stranded DNA, and inhibited telomerase activity by binding to G4 DNA [66,67]. Later, Zhang* et al.* demonstrated that 9-O-substituted berberine derivatives could induce and stabilize the anti-parallel G4 structure in the telomere DNA independently of the presence of metal cations [68]. Subsequently, Ma* et al.* designed and synthesized a series of 9-N-substituted berberine derivatives and investigated their interactions with NHE III_1_ DNA by means of electrophoretic mobility shift assay (EMSA), circular dichroism spectroscopy (CD), fluorescence resonance energy transfer-melting (FRET-melting) method, polymerase chain reaction-stop assay (PCR-stop assay), competition dialysis method, cell proliferation assay and reverse transcription-polymerase chain reaction (RT-PCR). All the results showed that the 9-N-substituted berberine derivatives could down-regulate the transcription of *c-myc* in cancer cell line by selectively stabilizing the formation of intramolecular parallel G4 in *c-myc* DNA [69].

Then, the same research group, by adding a pyridine ring and an amino group into berberine, improved the binding ability and selectivity towards G4 DNA in comparison with the previously reported 9-N-substituted berberine derivatives [70]. The synthesized quinoline benzo [5,6] dihydroisoquindolium compounds also showed good inhibition against *c-myc* transcription in tumor cells only.

More recently, Bournine* et al.* reported the anti-tumoral activity of *Glaucium flavum* root alkaloid extract against human cancer cells,* in vitro* and* in vivo*, highlighting protopine as the principal alkaloid identified by HPLC-DAD. Finally, the authors demonstrated a specific anticancer effect of *Glaucium flavum* root extract against breast cancer cells, which can be, at least in part, attributed to the other identified alkaloid bocconoline [71]. Recent ESI-MS analyses, performed by Cui* et al.* [72], reported three natural flexible cyclic molecules, tetrandrine, fangchinoline and cepharanthine, which can bind to the intermolecular and intramolecular RNA G4s from *r*(UAGGGUUAGGGU) and *r*(UAGGGUUAGGGUUAGGGUUAGGGU) sequences with high affinity.

All these studies highlight that the literature on alkaloids is growing rapidly. Researchers are persistently attempting to decode the many secrets surrounding alkaloids, since they believe that drugs developed from alkaloids or by using natural models of these compounds could help in the search for future cures to serious diseases, such as cancer or AIDS [73].

With respect to anticancer activity, we reported above many biophysical and biochemical studies which have shown that the natural benzophenantridine alkaloid berberine and its derivatives bind to human telomeric G4 structures, the *c-myc* oncogene and other biologically relevant G-rich sequences [67,69,74]. The interest toward this scaffold and G4 target lead Bazzicalupi* et al.* to solve the first crystal structure of human telomeric DNA in complex with the natural alkaloid berberine by X-ray diffraction method [75]. It was observed the presence of two berberine molecules in the two binding sites, directly interacting with each tetrad.

It is widely known that most of the G4-interactive compounds are planar molecules with multiple condensed rings that intercalate between the G-tetrad and the flanking residues of the pre-formed “drug binding pockets” [3,76,77,78,79].

Although there is a growing list of molecular structures reported for G4s formed in gene promoters, the structures of their drug complexes have been more difficult to obtain. In 2011 Dai* et al.* reported the in solution structure of a novel 2:1 complex between a quindoline, a derivative of the natural product cryptolepine, and a major G4 found in the *c-myc* promoter, using the sequence Pu22, in which G14 and G23 are mutated to thymines to isolate the single major conformation [80]. Thus, starting from these structural evidences and following the computational approach of our previous experiences [81,82], we performed a high throughput *in silico* screening of commercially available alkaloid compounds databases by means of a structure-based approach through docking experiments followed by molecular dynamics simulations against the human telomeric sequence *d*[AG_3_(T_2_AG_3_)_3_] and the *c-myc* promoter structure. We identified 69 best hits associated to an improved theoretical binding affinity with respect to that of the active set. Among them, a berberine derivative, already known to remarkably inhibit the telomerase activity, was related to a better theoretical affinity* versus* *c-myc*.

## 2. Results and Discussion

### 2.1. Structure-Based Virtual Screening of a Database Containing Natural Alkaloids and Berberine Analogues

**Figure 1:**
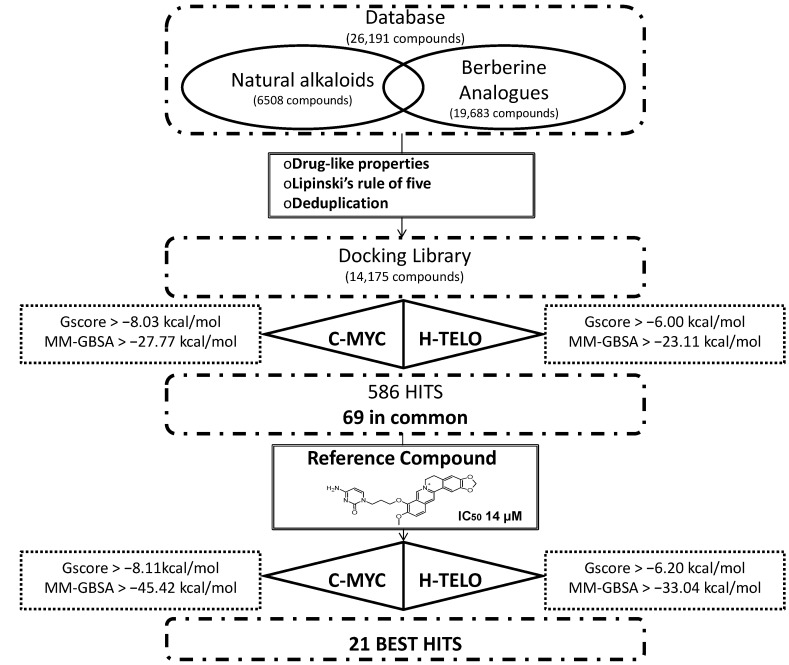
Summary of the theoretical flow chart adopted for the identification of 21 best hits starting from a natural database.

In order to identify new natural DNA G4 binders, a structure-based virtual screening of a database containing 26,191 compounds and the application of a series of filters led us to finally identify 21 best hits that showed an improved theoretical binding affinity with respect to a reference berberine derivative (Figure 1).

The database used, containing 26,191 molecules, was built combining libraries of alkaloids and berberine analogues. Subsequently, the filter for drug-like properties, the Lipinski’s rule of five and the deduplication led us to globally consider 14,175 compounds. The human telomeric (*h-telo)* and *c-myc* promoter oncogene DNA G4s, respectively with PDB codes 3R6R [75] and 2L7V [80], were used to perform the structure-based virtual screening procedure. In particular, Glide Standard Precision (SP) docking simulations [83] were carried out to evaluate the recognition of the 14,175 compounds against DNA G4s. For evaluating the accuracy and reliability of our docking procedure, we firstly performed Glide SP redocking calculations of berberine and quindoline against 3R6R (*h-telo*) and 2L7V (*c-myc*) receptors, respectively, in order to geometrically reproduce the experimental data. The obtained poses with the related RMSd values are shown in Figure S1 of the Supplementary Materials.

The best Glide pose for *h-telo* and *c-myc* DNA G4 receptors was considered and subjected to further analysis. Specifically, the Molecular Mechanics/Generalized Born Surface Area (MM-GBSA) binding free energy [84] was computed. In order to set an energy cut-off applied to choose the best scored ligands, the same docking protocol and binding free energy analysis was carried out for the 4 active compounds quarfloxine, triazine derivative 12459, quindoline and berberine [85,86,87,88] (Table 1), chosen on the basis of their known activity towards the target.

Based on the active compounds free energy of binding, we decided to choose the lowest value of both scoring function on both receptors. Specifically, based on the Glide score (G-score), 1701 compounds with a value lower than −6.00 kcal/mol for *h-telo* receptor and 378 ligands with a value lower than −8.03 kcal/mol for the *c-myc* were selected. All these compounds were filtered on the basis of their MM/GBSA free energy of binding. Within the cut-off value lower than −23.11 kcal/mol for *h-telo* and lower than −27.77 kcal/mol for *c-myc*, 385 and 201 compounds were selected, respectively. In order to further filter them, starting from the obtained 586 compounds, we considered only the 69 ligands with a good binding affinity on both *h-telo* and *c-myc* G4s, as reported in Table S1 of the Supplementary Materials. Among these compounds, 54 belong to the berberine derivative class, while the remaining 15 are alkaloids.

Among the 69 best hits, several were associated to antitumor activity, some were Rac GTPases inhibitors and others reported no documented activity (Table S1 of the Supplementary Materials). Interestingly, our virtual screening approach identified two ligands (CID IDs 71165335 and 71476670) found to behave as telomerase inhibitors and actually patent pending. Therefore, even if such a result could represent a validation of our theoretical method, we preferred to discard them from our hits selection, that has been decreased to 67 molecules.

**Table 1:** The best Glide score (G-score) and MM-GBSA values obtained after docking simulations of active compounds against *h-telo* (PDB 3R6R) and *c-myc* (PDB 2L7V) G4 receptors. The energy G-scores and MM-GBSA energy values are expressed in kcal/mol.

Ligands	Chemical Structure	*h-telo*		*c-myc*	
		G-Score	MM-GBSA	G-Score	MM-GBSA
Quarfloxine	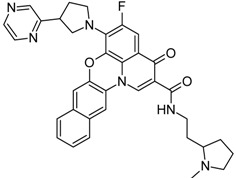	−7.67	−32.28	−8.98	−27.77
Triazine derivative 12459	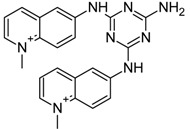	−7.49	−34.17	−8.90	−56.60
Quindoline	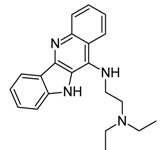	−7.00	−23.11	−9.35	−43.00
Berberine	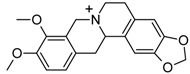	−6.00	−26.42	−8.03	−33.93

**Figure 2:**
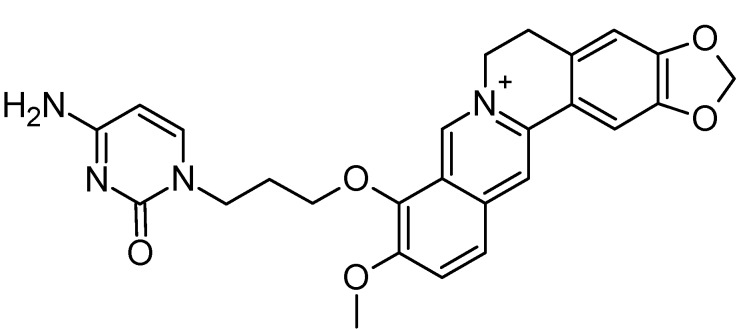
The 2D chemical representation of the reference berberine derivative.

However, since among all the selected compounds only one was found with a documented activity to work as binder of the human telomeric G4 DNA sequence [89], we focused our attention on this ligand (CID ID 44583341), that is reported in Figure 2.

In particular, this 9-*O*-substituted berberine derivative, containing an aza-aromatic terminal group, was intensively evaluated by CD and fluorescence spectroscopy, molecular modeling, FRET-melting assay, PCR stop assay and TRAP assay. The results from biophysical and biochemical assay indicated that the compound possessed much stronger stabilizing effect on the telomeric G4 with respect to berberine [89].

**Figure 3:**
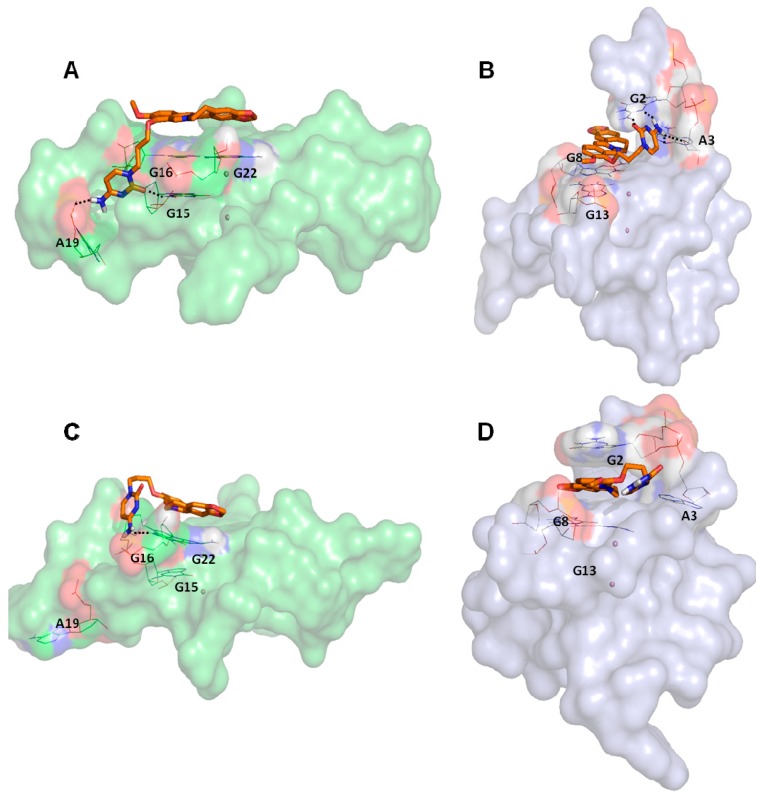

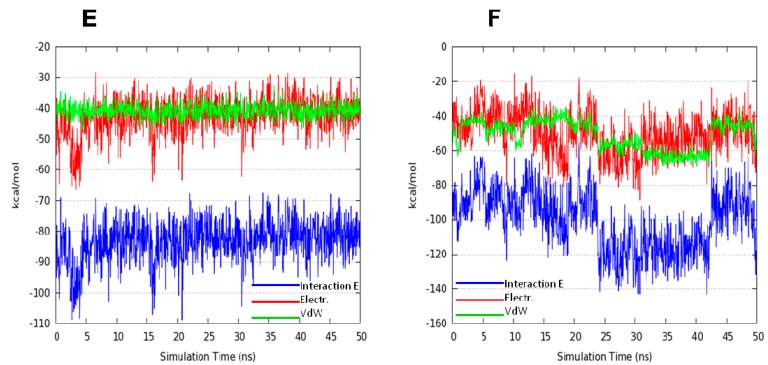
Representation of the best pose after the docking simulations of the berberine derivative against (**A**) *h-telo* and (**B**) *c-myc* G4 structures. Representation of the most populated structure after the molecular dynamics simulations of the complexes between the berberine derivative and (**C**) *h-telo* and (**D**) *c-myc* G4 structures. The ligand is shown in orange carbon sticks, the nucleobases involved in hydrogen bonds, reported as black dashed lines, are represented in wireframe, while the coordinating cations are shown as purple spheres. Monitoring of the energy profile after 50 ns of MDs in terms of Interaction Energy (blue), electrostatic (red) and Van der Waal’s (green) contributions, in (**E**) *h-telo* and (**F**) *c-myc* G4 complexes. The energy values are expressed in kcal/mol.

The current growing interest in the G4 DNA structures of promoter regions [90,91], in particular* versus* *c-myc*, led us to analyze the docking poses obtained on *h-telo* and *c-myc* receptors. With the aim to highlight the differences in the recognition of the used DNA G4 folds, we performed a contact analysis evaluating the interactions in terms of hydrogen bonds and Van der Waal’s contacts by means of Maestro graphical interface (Maestro Graphics User Interface, ver. 9.7, Schrödinger LLC, New York, NY, USA, 2014) [92,93] (Table 2). The docking best pose of the reference berberine derivative against the *h-telo* receptor showed its planar surface involved in several end stacking interactions with the G4, G15, G16, G20, G22, T17 and A19 nucleobases. However, due to the presence of a side chain, the ligand was able to establish two hydrogen bonds with the guanine at position 15 and the adenine at position 19 (Figure 3A). Interestingly, in the *c-myc* G4 recognition, the compound presented an improved binding affinity and a better interaction pattern compared to *h-telo*. In particular, the berberine derivative was well stabilized by four hydrogen bonds established with the bases G2 and A3, as shown in Figure 3B. Moreover, the compound was also involved in several Van der Waal’s contacts (more than twofold with respect to the *h-telo* G4), especially with the guanines at position 13 and 8 (Table 2). Using the berberine derivative as reference compound, its G-score and MM-GBSA values were adopted as additional filters of the 69 best hits. In particular, 52 compounds, with *h-telo* G-score values lower than −6.20 kcal/mol, and 57 ligands, with *c-myc* G-score values lower than −8.11 kcal/mol, for the *c-myc*, were selected. Considering the MM-GBSA binding free energies, we decided on 17 and 8 compounds, respectively, for *h-telo* (<−33.04 kcal/mol) and *c-myc* (<−45.42 kcal/mol), 4 of which are common (Table S1 in the Supplementary Materials).

**Table 2:** Global number of contacts and free binding energy values, expressed in kcal/mol, of the reference berberine derivative complexed to 3R6R (*h-telo*) and 2L7V (*c-myc*) models, obtained with the Glide SP approach. HB and GC indicate, respectively, the hydrogen bonds and the good contacts established by the ligand.

h-telo				c-myc			
HB	GC	G-Score	MM-GBSA	HB	GC	G-Score	MM-GBSA
2	148	−6.20	−33.04	4	331	−8.11	−45.42

In order to further refine the docking results of our reference compound, the best poses on *h-telo* and *c-myc* receptors were subjected to molecular dynamics simulations.

### 2.2. Molecular Dynamics Simulation (MDs)

With the aim to explain the binding stability of our reference compound and to clarify the role of its side chain, molecular dynamics simulations (MDs) were performed using NAMD program [94] with the parameters reported in the experimental section. After 50 ns of MDs of *h-telo* and *c-myc* complexes, the ligand was found to stabilize the G4 core without unfolded structure. Specifically, the Root Mean Square deviation (RMSd) calculated on the guanines core showed the slightly lower average value (equal to 1.20 Å) for the *c-myc* complex, with respect to that of *h-telo* (RMSd equal to 1.37 Å). Moreover, to better characterize the interactions between the ligand and both G4 receptors, we evaluated the non-bonded interaction energies in terms of electrostatic and Van der Waal’s single contributions, using the NAMD Energy Plugin [94]. We observed that, according to the theoretical docking data, the average Interaction Energy value, calculated after 50 ns of MDs, was equal to −82.70 kcal/mol and −100.54 kcal/mol for *h-telo* and *c-myc*, respectively.

In particular, it was evident that in *c-myc* complex, after approximately 25 ns, the Van der Waal’s term was associated to the major contribution, accordingly to a sandwiched binding mode between the berberine core and the guanines nucleobases at positions 2, 3, 8 and 13, respectively. Conversely, for the *h-telo* receptor, the electrostatic and Van der Waal’s energetic contributions were almost equivalent (Figure 3E,F) and the complex stability seemed to be related to the formation of only one hydrogen bond between the aza-aromatic side chain of the ligand and the G16 nucleobase, already involved in a stacking contact.

## 3. Experimental Section

### 3.1. Database Preparation and Filtering Procedure

Both natural alkaloids (6508) and berberine analogues (similarity equal to 80%, 19,683) libraries were downloaded from PubChem [95] and merged, thus obtaining overall a database of 26,191 compounds. All the molecules included in such database were prepared considering the ionization state at physiological 7.4 pH and then energy minimized using the MMFFs force field as implemented in the LigPrep platform ver. 2.9 [96] of Maestro. Finally, they were filtered basing on their drug-like properties as it has been addressed by the Lipinski’s rule of 5 [97] and the duplicated structures were removed. A total amount of 14,175 compounds successfully passed these filters and were subjected to the further molecular recognition process.

### 3.2. Receptors Preparation

The 3D starting structures of the *h-telo* [AG_3_T_2_AG_3_T_2_AG_3_T_2_AG_3_] and *c-myc* promoter oncogene [TGAG_3_TG_3_TAG_3_TG_3_TA_2_] DNA G4 sequences were downloaded from the Protein Data Bank website [98] with the PDB codes 3R6R [75] and 2L7V [80], respectively. After removing the ligands, both receptors were refined and optimized using the Protein Preparation Wizard tool [99]. Hydrogen atoms were added and the geometry of all the hetero groups was corrected.

### 3.3. Receptor Grid Generation and Docking Simulations

Glide grids were generated by Receptor Grid Generation as implemented in Glide ver. 6.2 [83]. Grid box of both receptors was centered considering the x, y, z coordinates of the G-tetrads centroid and set up wide enough to include the entire macromolecules. The binding affinity for each of the 14175 compounds against *h-telo* and *c-myc* G4 sequences was predicted using Glide Standard Precision (SP) protocol, generating ten poses for each ligand. Moreover, the binding free energy was computed by Molecular Mechanics/Generalized Born Surface Area (MM-GBSA) method, VSGB 2.0 continuum dielectric model as solvent model [84], as implemented in Prime [100]. The same docking protocol was adopted for a set of 4 active ligands [85,86,87,88].

### 3.4. MD Simulation Protocol

In order to further clarify the binding stability of the reference compound (CID: 44583341) and elucidate the role of its side chain, standard MD simulations were performed with NAMD code ver. 2.9 [94] on both *h-telo* and *c-myc* sequences. The *parm99* Amber force field, including the recent nucleic acids *parmbsc0* parameters [101], was used. Complexes were then placed in a 12.0 Å layer cubic water box using the TIP3P explicit water model. K^+^ cations were added to neutralize the net charge. The SHAKE algorithm was applied to constraint bonds involving hydrogen atoms. A 2 fs integration time step was considered. Ligand charges were computed using the restrained electrostatic potential (RESP) fitting procedure [102]. The ESP was first calculated by means of Jaguar application ver. 8.3 [103] using a 6-31G* basis set at the Hartree-Fock level of theory. Finally, the RESP charges were calculated by means of the Antechamber module [104].

The system was thus subjected to a double minimization step using the conjugate gradient algorithm in the following conditions: (i) minimization of water molecules and ions, keeping all the solute fixed (2000 steps); (ii) minimization of the entire system, without any restriction (2000 steps). The system was thus equilibrated at 300 K through 1ns in NVT and 1 atm through 1 ns in NPT ensembles before performing the MD production run of 50 ns in the NPT ensemble.

## 4. Conclusions

In this work we carried out a high throughput *in silico* screening of commercially available alkaloids databases by means of a structure-based approach based on docking experiments and molecular dynamics simulations against *h-telo* and *c-myc* G4 structures. We identified 69 best hits associated to an improved theoretical binding affinity with respect to that of the active set. Among them, we found a berberine derivative, already known to remarkably inhibit the telomerase activity, related to a better theoretical affinity* versus* *c-myc*. This compound, considered as reference ligand, was used to apply a further filter to identify a series of new hits from the used natural database. In particular, the 21 best hits that showed an improved theoretical binding affinity with respect to this reference compound, are currently under experimental investigations in order to complete their biophysical and biological profiles. Moreover, our computational protocol could be applied to other libraries of molecules without a known activity on *h-telo* and *c-myc* DNA G4s in order to identify the most promising ones that will be optimized against this emergent target.

## Supplementary Materials

Supplementary materials can be accessed at: http://www.mdpi.com/1420-3049/20/01/0206/s1.
