# Supplementary material for: Structure-Based Virtual Screening of Novel Natural Alkaloid Derivatives as Potential Binders of h-telo and c-myc DNA G-Quadruplex Conformations
Source: Molecules. 2014 Dec 24;20(1):206–23. doi: 10.3390/molecules20010206 (PMC6272608; doi:10.3390/molecules20010206)
Supplement: Supplementary file 1 [file molecules-20-00206-s001.pdf]

Supplementary Materials

Table of Contents

Table S1. Table of 2D chemical structure, G-score and MM-GBSA values of the 69 best hits obtained after docking simulations against *h-telo* and *c-myc* G4 structures.

Figure S1. Best redocking poses and RMSd values obtained using the Glide-SP algorithm for berberine and quindoline against 3R6R (*h-telo*) and 2L7V (*c-myc*) receptors, respectively. Redocked poses are displayed as green carbon sticks, while crystallographic conformation is shown as cyan carbon sticks.

Table S1. 2D chemical structure, G-score and MM-GBSA values of the 69 best hits, obtained after docking simulations against *h-telo* and *c-myc* G4 structures. The CID ID indicates the identifier for the test compounds; G-score and MM-GBSA values are expressed in kcal/mol. The ligands marked with the asterisk belong to the final selection of compounds currently under experimental investigations.

**Berberine Analogues**						**Anticancer Activity and if Available Target**
		h-telo		***c-myc***		
**CID ID**	**2D Chemical Structure**	**G-Score**	**MM/GBSA**	**G-Score**	**MM/GBSA**	
**10269105 ***	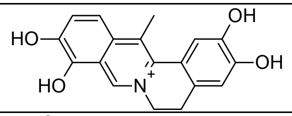	−6.77	−53.64	−8.60	−43.71	Antitumor [1]
**71496902 ***	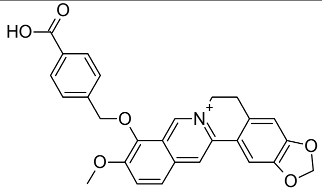	−6.88	−46.76	−8.44	−44.15	-
**10133114**	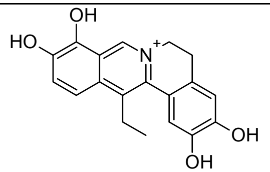	−6.13	−45.92	−8.05	−43.18	Antitumor [1]
**68426959**	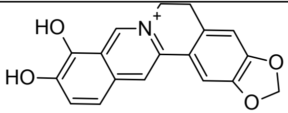	−6.15	−43.18	−8.32	−38.01	Phospholipase A2 inhibitor [2]
**21417729 ***	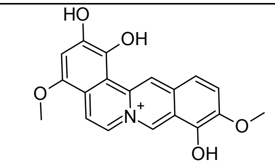	−7.36	−42.23	−8.27	−36.21	Antitumor [3]
**9880653 ***	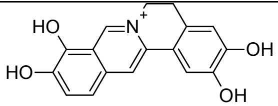	−6.37	−40.41	−9.06	−48.23	Rac GTPases inhibitor [4]

Table S1. *Cont.*

Berberine Analogues						Anticancer Activity and if Available Target
		h-telo		*c-myc*		
**CID ID**	**2D Chemical Structure**	**G-Score**	**MM/GBSA**	**G-Score**	**MM/GBSA**	
**49870985 ***	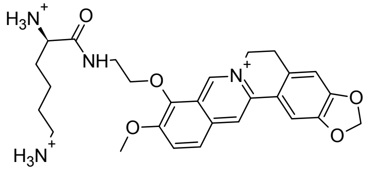	−10.05	−39.87	−9.49	−49.12	Calf thymus (CT) DNA binder [5]
**21417482 ***	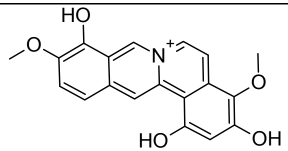	−6.83	−38.67	−8.38	−38.71	Antitumor [3]
**11618402 ***	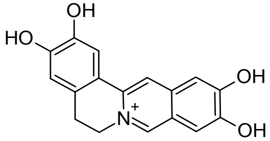	−6.43	−38.42	−9.34	−54.37	Rac GTPases inhibitor [4]
**21417576 ***	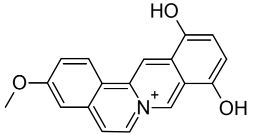	−6.59	−36.50	−8.89	−32.52	Antitumor [6]
**3939583 ***	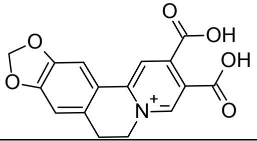	−7.17	−35.16	−8.94	−41.29	-
**58064859 ***	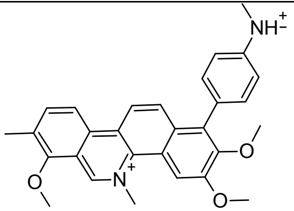	−6.46	−35.13	−8.59	−34.37	-

Table S1. *Cont.*

Berberine Analogues						Anticancer Activity and if Available Target
		h-telo		*c-myc*		
**CID ID**	**2D Chemical Structure**	**G-Score**	**MM/GBSA**	**G-Score**	**MM/GBSA**	
**71165335**	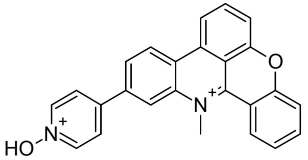	−7.30	−34.72	−9.73	−51.64	Telomerase inhibitor [7]
**10358881** *	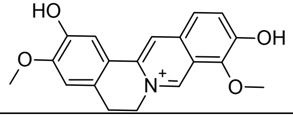	−6.03	−34.52	−8.67	−49.48	-
**21417681** *	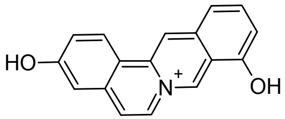	−6.55	−34.47	−9.22	−32.79	-
**29146231** *	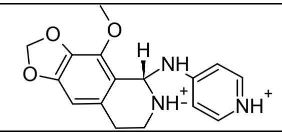	−7.68	−34.26	−9.06	−38.74	-
**68071758** *	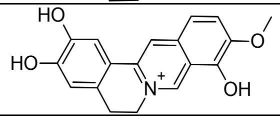	−7.21	−34.04	−8.97	−49.92	-
**25164618** *	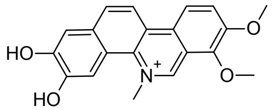	−6.29	−33.82	−9.01	−43.44	Antitumor [8]
**72323** *	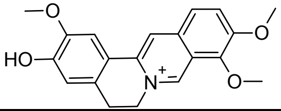	−6.29	−33.40	−8.71	−39.71	Antitumor [9]

Table S1. *Cont.*

**Berberine Analogues**						**Anticancer Activity and if Available Target**
		h-telo		***c-myc***		
**CID ID**	**2D Chemical Structure**	**G-Score**	**MM/GBSA**	**G-Score**	**MM/GBSA**	
**21417634**	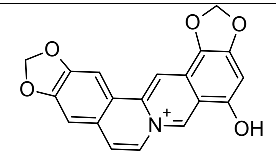	−6.06	−33.30	−8.06	−31.81	-
**44583341 ***	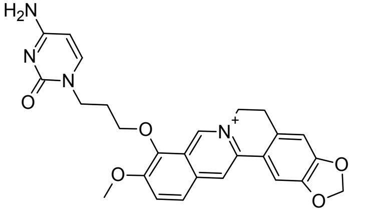	−6.20	−33.04	−8.11	−45.42	G4 binder [10,11]
**53343538**	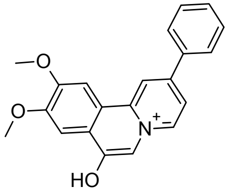	−6.71	−32.78	−8.52	−34.01	-
**21417462 ***	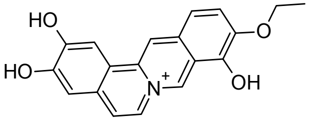	−6.65	−32.13	−9.14	−49.69	-
**70848286**	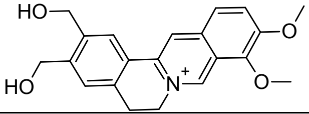	−6.36	−32.10	−8.56	−41.83	-
**66602686**	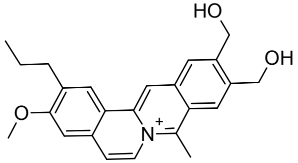	−6.53	−31.70	−8.87	−34.34	-

Table S1. *Cont.*

Berberine Analogues						Anticancer Activity and if Available Target
		h-telo		*c-myc*		
**CID ID**	**2D Chemical Structure**	**G-Score**	**MM/GBSA**	**G-Score**	**MM/GBSA**	
**45483073**	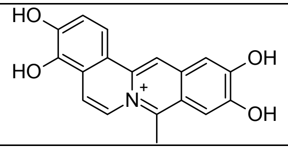	−6.83	−31.44	−9.22	−43.96	Rac GTPases inhibitor [12]
**24839791**	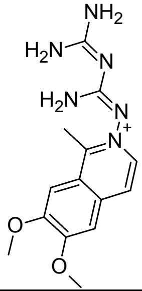	−6.15	−31.12	−8.49	−41.03	-
**21417600 ***	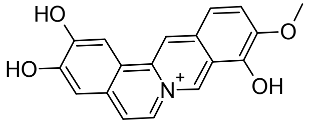	−6.98	−30.95	−8.86	−48.51	-
**24200207**	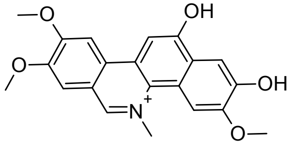	−6.09	−30.81	−9.13	−38.58	Antitumor [13]
**21417591 ***	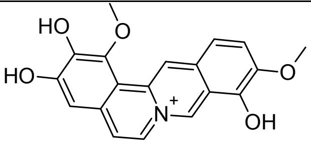	−6.86	−30.42	−8.29	−47.09	-

Table S1. *Cont.*

Berberine Analogues						Anticancer Activity and if Available Target
		h-telo		*c-myc*		
**CID ID**	**2D Chemical Structure**	**G-Score**	**MM/GBSA**	**G-Score**	**MM/GBSA**	
**21417448**	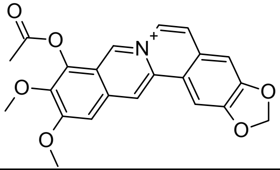	−6.32	−30.22	−8.10	−28.79	-
**161372**	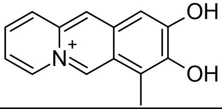	−6.20	−30.20	−8.58	−40.49	Antitumor [14]
**21417712**	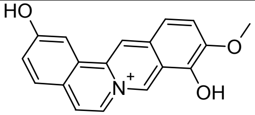	−7.25	−30.11	−8.47	−35.08	-
**21417707**	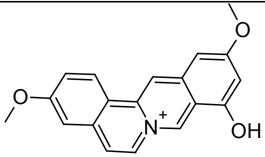	−6.33	−30.10	−8.18	−31.68	-
**71476670**	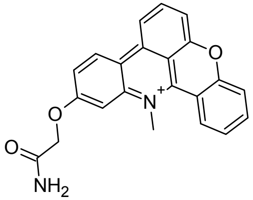	−6.38	−29.98	−8.98	−34.24	Telomerase inhibitor [7]
**21417594**	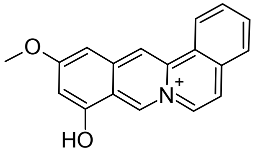	−6.50	−29.90	−8.22	−31.63	-

Table S1. *Cont.*

Berberine Analogues						Anticancer Activity and if Available Target
		h-telo		*c-myc*		
**CID ID**	**2D Chemical Structure**	**G-Score**	**MM/GBSA**	**G-Score**	**MM/GBSA**	
**21417589**	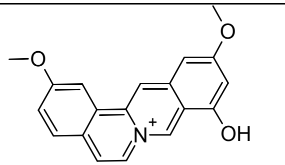	−6.30	−29.82	−8.27	−29.61	-
**25093287**	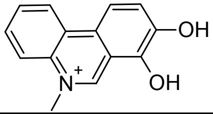	−6.28	−29.32	−8.27	−32.60	Bcl-XL Inhibitor [15]
**21417511**	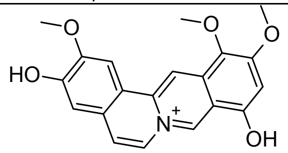	−6.25	−28.99	−8.08	−35.17	-
**21417657**	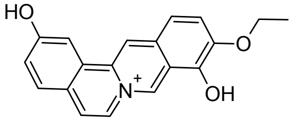	−6.84	−28.52	−8.18	−37.81	-
**21417566**	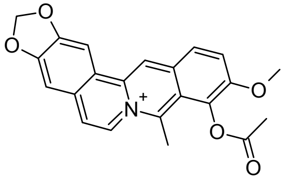	−6.13	−28.47	−8.03	−30.27	-
**68119699**	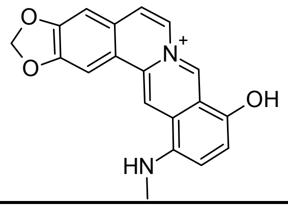	−6.07	−28.17	−8.22	−32.10	-

Table S1. *Cont.*

	Berberine Analogues					Anticancer Activity and if Available Target
		h-telo		*c-myc*		
**CID ID**	**2D Chemical Structure**	**G-Score**	**MM/GBSA**	**G-Score**	**MM/GBSA**	
**21417545**	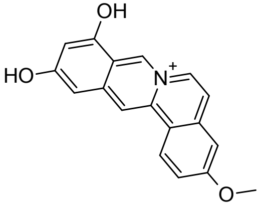	−6.82	−28.01	−8.84	−33.02	-
**24198806**	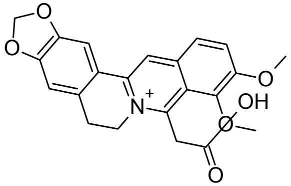	−6.53	−27.78	−8.24	−40.44	-
**21417717**	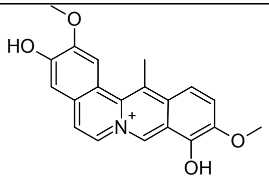	−6.11	−27.73	−8.81	−39.15	-
**24196957**	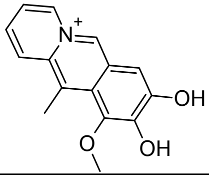	−6.00	−27.45	−8.65	−43.45	-
**68119998**	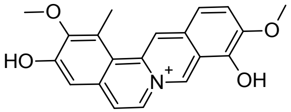	−6.25	−27.18	−8.03	−39.11	-
**21417624**	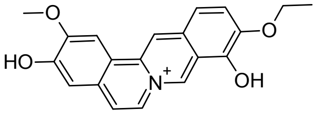	−6.05	−26.91	−8.99	−29.19	-

Table S1. *Cont.*

	Berberine Analogues					Anticancer Activity and if Available Target
		h-telo		*c-myc*		
**CID ID**	**2D Chemical Structure**	**G-Score**	**MM/GBSA**	**G-Score**	**MM/GBSA**	
**53343867**	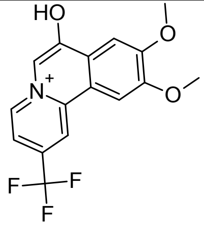	−6.39	−26.10	−8.05	−31.53	-
**53343540**	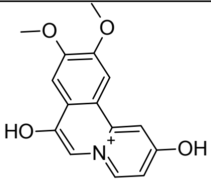	−6.70	−25.79	−8.46	−32.32	-
**53902617**	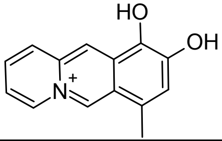	−6.21	−25.78	−8.71	−34.77	-
**53343542**	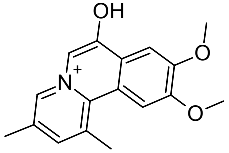	−6.43	−24.29	−8.28	−31.15	-
**25165620**	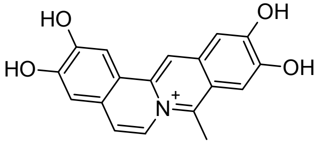	−6.51	−24.04	−9.61	−44.41	Rac GTPases inhibitors [12]

Table S1. *Cont.*

**Berberine Analogues**						
		h-telo		***c-myc***		**Anticancer Activity and if Available Target**
**CID ID**	**2D Chemical Structure**	**G-Score**	**MM/GBSA**	**G-Score**	**MM/GBSA**	
**53343544**	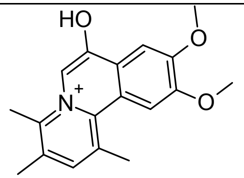	−6.13	−23.84	−8.29	−28.59	-
**Natural Alkaloids**						
		h-telo		***c-myc***		
**CID**	**2D Chemical structure**	**G-Score**	**MM/GBSA**	**G-Score**	**MM/GBSA**	
**53318152** *	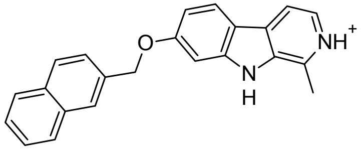	−6.29	−38.57	−8.07	−31.44	Antitumor [16]
**24828402**	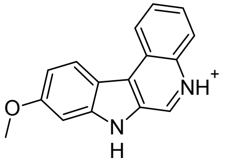	−6.10	−32.94	−8.30	−35.15	-
**25258753**	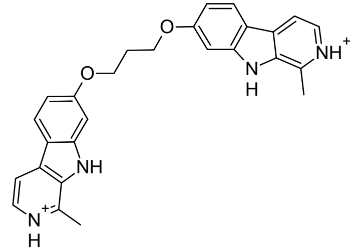	−6.84	−32.57	−8.33	−44.26	Antitumor [17]

Table S1. *Cont.*

Berberine Analogues						Anticancer Activity and if Available Target
		h-telo		*c-myc*		
**CID ID**	**2D Chemical Structure**	**G-Score**	**MM/GBSA**	**G-Score**	**MM/GBSA**	
**24951900**	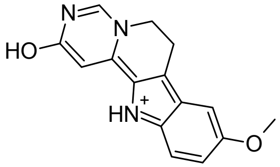	−8.21	−30.34	−8.03	−39.19	-
**10890876**	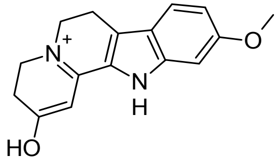	−6.76	−30.09	−8.17	−31.69	-
**12443570**	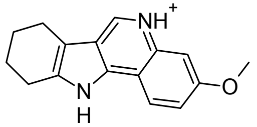	−6.48	−29.45	−8.09	−34.28	-
**20472807**	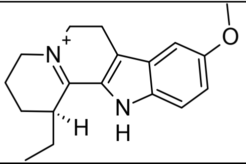	−6.38	−29.33	−8.31	−32.93	-
**53322103**	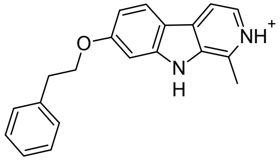	−6.22	−29.27	−8.15	−30.19	Antitumor [16,18]
**49801392**	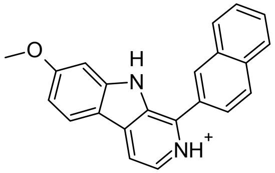	−6.31	−28.76	−8.17	−29.81	Antitumor [19]

Table S1. *Cont.*

Berberine Analogues						Anticancer Activity and if Available Target
		h-telo		*c-myc*		
**CID ID**	**2D Chemical Structure**	**G-Score**	**MM/GBSA**	**G-Score**	**MM/GBSA**	
**5137933**	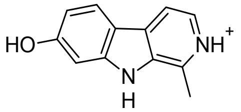	−6.83	−27.37	−8.21	−32.46	Antitumor [5,20]
**21362738**	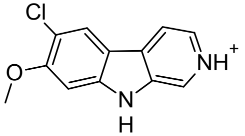	−6.10	−27.09	−8.57	−31.83	IκB kinase inhibitor [21]
**3373692**	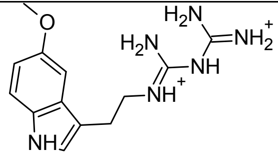	−9.11	−25.93	−8.28	−31.47	-
**54575584**	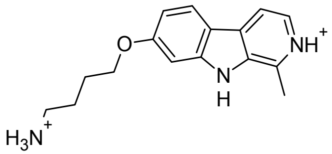	−6.90	−25.78	−10.02	−32.15	Haspin kinase inhibitor [22,23]
**607775**	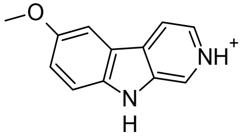	−7.18	−24.11	−8.37	−28.53	IKK inhibitor [24]
**5314681**	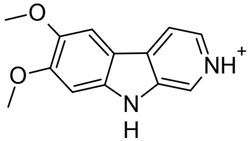	−6.46	−23.95	−8.65	−31.72	-

Figure S1. Best redocking poses and RMSd values obtained using the Glide-SP algorithm for berberine and quindoline against 3R6R (*h-telo*) and 2L7V (*c-myc*) receptors, respectively. Redocked poses are displayed as green carbon sticks, while crystallographic conformation is shown as cyan carbon sticks.

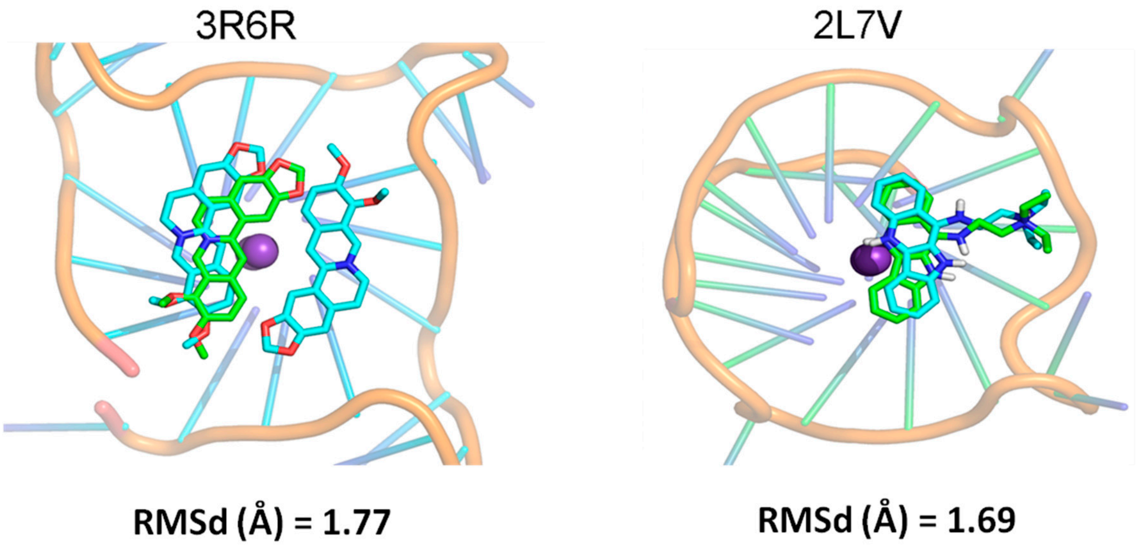

References

1. Sawa, Y.; Ikekawa, T. 13-Substituted Palmatine Derivatives. JP50070398A, 11 June 1975.
2. Naveen Chandra, D.; Abhilash, J.; Prasanth, G.K.; Sabu, A.; Sadasivan, C.; Haridas, M. Inverted binding due to a minor structural change in berberine enhances its phospholipase A2 inhibitory effect. *Int. J. Biol. Macromol.* **2012**, *50*, 578–585.
3. Sawa, Y.; Ikekawa, T. 9-Hydroxy-10-methoxydibenzo[a,g]quinolizinium Salts. JP50148397A, 27 November 1975.
4. Beausoleil, E.; Chauvignac, C.; Taverne, T.; Lacombe, S.; Pognante, L.; Leblond, B.; Pallares, D.; De Oliveira, C.; Bachelot, F.; Carton, R.; *et al*. Structure-activity relationship of isoform selective inhibitors of Rac1/1b GTPase nucleotide binding. *Bioorg. Med. Chem. Lett.* **2009**, *19*, 5594–5598.
5. Abe, A.; Kokuba, H. Harmol induces autophagy and subsequent apoptosis in U251MG human glioma cells through the downregulation of survivin. *Oncol. Rep.* **2013**, *29*, 1333–1342.
6. Sawa, Y. Dibenzo-Shaped Clip on a to g Chinoliziniumverbindungen Square Peg, Process for Their Production and Drug-Containing. DE2520524A1, 27 November 1975.
7. Cousin, D.; Frigerio, M.; Hummersone, M.G. Fused Pentacyclic Anti-Proliferative Compounds. WO2012175991A1, 27 December 2012.
8. Hanaoka, M.; Ekimoto, H.; Kobayashi, F.; Irie, Y.; Takahashi, K.; Suzuki, M.; Nakanishi, T.; Kogawa, O.; Ishikawa, K. Process for Preparing Benzo [*c*] Phenanthridinium Derivatives, Novel Compounds Prepared by Said Process, and Antitumor Agents. U.S. Patent US5747502A, 5 May 1998.
9. Kumari, S.; Kaladhar, D.S. V.G. K.; Sandeep Solmon, K.; Malla, R.R.; Kishore, G. Anti-proliferative and metastatic protease inhibitory activities of protoberberines: An in silico and *in vitro* approaches. *Process Biochem. (Oxford, UK)* **2013**, *48*, 1565–1571.

10. Ebrahimi, M.; Khayamian, T. Interactions of G-quadruplex DNA binding site with berberine derivatives and construct a structure-based QSAR using docking descriptors. *Med. Chem. Res.* **2014**, *23*, 1327–1339.
11. Ma, Y.; Ou, T.-M.; Tan, J.-H.; Hou, J.-Q.; Huang, S.-L.; Gu, L.-Q.; Huang, Z.-S. Synthesis and evaluation of 9-*O*-substituted berberine derivatives containing aza-aromatic terminal group as highly selective telomeric G-quadruplex stabilizing ligands. *Bioorg. Med. Chem. Lett.* **2009**, *19*, 3414–3417.
12. Leblond, B.; Beausoleil, E.; Chauvignac, C.; Taverne, T.; Picard, V.; De Oliveira, C.; Schweighoffer, F. Compounds and Methods for Modulating Rho Gtpases. WO2009007457A2, 15 January 2009.
13. Olugbade, T.A.; Waigh, R.D. Synthetic benzo[c]phenanthridines with antileukemic activity in mice. *Pharm. Sci.* **1996**, *2*, 259–264.
14. Missirlis, E.; Karakiulakis, G.; Maragoudakis, M.E. Antitumor effect of GPA1734 in rat Walker 256 carcinoma. *Investig. New Drugs* **1990**, *8*, 145–147.
15. Bernardo, P.H.; Wan, K.-F.; Sivaraman, T.; Xu, J.; Moore, F.K.; Hung, A.W.; Mok, H.Y.K.; Yu, V.C.; Chai, C.L.L. Structure-Activity Relationship Studies of Phenanthridine-Based Bcl-XL Inhibitors. *J. Med. Chem.* **2008**, *51*, 6699–6710.
16. Frederick, R.; Masereel, B.; Reniers, J.; Wouters, J.; Bruyere, C.; Kiss, R. Beta Carboline Derivatives Useful in the Treatment of Proliferative Disorders. WO2011161256A1, 29 December 2011.
17. Desmaeele, D.; Auclair, C.; Zouhiri, F.; Polard, V.; Maksimenko, A. Dimers of Harmol or of Its Derivatives and Uses Thereof. EP2050747A1, 22 April 2009.
18. Frederick, R.; Bruyere, C.; Vancraeynest, C.; Reniers, J.; Meinguet, C.; Pochet, L.; Backlund, A.; Masereel, B.; Kiss, R.; Wouters, J. Novel Trisubstituted Harmine Derivatives with Original in Vitro Anticancer Activity. *J. Med. Chem.* **2012**, *55*, 6489–6501.
19. Buolamwini, J.K. 1-Aryl or 1- Heteroaryl-Pyrido(B)indoles and Uses Thereof. U.S. Patent US20130131070A1, 23 May 2013.
20. Yu, L.; Hu, L.; Tang, L.; Liu, J.; Liu, Z. Application of Harmol to Manufacture of Antitumor Agents. CN103417536A, 4 December 2013.
21. Ritzeler, O.; Castro, A.; Grenier, L.; Soucy, F.; Hancock, W.W.; Mazdiyasni, H.; Palombella, V.; Adams, J. Substituted Beta-Carbolines. EP1209158A1, 29 May 2002.
22. Cuny, G.D.; Ulyanova, N.P.; Patnaik, D.; Liu, J.-F.; Lin, X.; Auerbach, K.; Ray, S.S.; Xian, J.; Glicksman, M.A.; Stein, R.L.; *et al.* Structure-activity relationship study of beta-carboline derivatives as haspin kinase inhibitors. *Bioorg. Med. Chem. Lett.* **2012**, *22*, 2015–2019.
23. Higgins, J.; Patnaik, D.; Ulyanova, N.; Stein, R.L.; Xian, J.; Glicksman, M.; Cuny, G.D. Beta-Carbolines as Inhibitors of Haspin and DYRK Kinases and Their Preparation, Pharmaceutical Compositions and Use in the Treatment of Diseases. WO2011133795A2, 27 October 2011.
24. Castro, A.C.; Dang, L.C.; Soucy, F.; Grenier, L.; Mazdiyasni, H.; Hottelet, M.; Parent, L.; Pien, C.; Palombella, V.; Adams, J. Novel IKK inhibitors: β-carbolines. *Bioorg. Med. Chem. Lett.* **2003**, *13*, 2419–2422.
